# A Natural mtDNA Polymorphism in Complex III Is a Modifier of Healthspan in Mice

**DOI:** 10.3390/ijms20092359

**Published:** 2019-05-13

**Authors:** Misa Hirose, Axel Künstner, Paul Schilf, Anna Katharina Tietjen, Olaf Jöhren, Patricia Huebbe, Gerald Rimbach, Jan Rupp, Markus Schwaninger, Hauke Busch, Saleh M. Ibrahim

**Affiliations:** 1 Luebeck Institute of Experimental Dermatology, University of Luebeck, 23562 Luebeck, Germany; Misa.Hirose@uksh.de (M.H.); Paul.Schilf@uksh.de (P.S.); annakatharinatietjen@gmail.com (A.K.T.); 2 Luebeck Institute of Experimental Dermatology and Institute for Cardiogenetics, University of Luebeck, 23562 Luebeck, Germany; axel.kuenstner@uni-luebeck.de; 3 Center of Brain, Behavior & Metabolism, University of Luebeck, 23562 Luebeck, Germany; olaf.joehren@uni-luebeck.de; 4 Institute of Human Nutrition and Food Science, University of Kiel, 24098 Kiel, Germany; huebbe@foodsci.uni-kiel.de (P.H.); rimbach@foodsci.uni-kiel.de (G.R.); 5 Department of Infectious Disease and Microbiology, University of Luebeck, 23562 Luebeck, Germany; Jan.Rupp@uksh.de; 6 Institute of Experimental and Clinical Pharmacology and Toxicology, University of Luebeck, 23562 Luebeck, Germany; markus.schwaninger@pharma.uni-luebeck.de; 7 Luebeck Institute of Experimental Dermatology, Institute for Cardiogenetics and Center for research of inflammatory skin disease (CRIS), University of Luebeck, 23562 Luebeck, Germany; hauke.busch@uni-luebeck.de; 8 Luebeck Institute of Experimental Dermatology and Center for research of inflammatory skin disease (CRIS), University of Luebeck, 23562 Luebeck, Germany

**Keywords:** mitochondrial DNA polymorphisms, conplastic mouse strains, complex III, mitochondrially encoded cytochrome b gene, *mt-Cytb*, age-related diseases, middle-aged obesity, healthspan, gut microbiota

## Abstract

In this study, we provide experimental evidence that a maternally inherited polymorphism in the mitochondrial cytochrome b gene (*mt-Cytb*; m.15124A>G, Ile-Val) in mitochondrial complex III resulted in middle-aged obesity and higher susceptibility to diet-induced obesity, as well as age-related inflammatory disease, e.g., ulcerative dermatitis, in mice. As a consequence of the gene variation, we observed alterations in body composition, metabolism and mitochondrial functions, i.e., increased mitochondrial oxygen consumption rate and higher levels of reactive oxygen species, as well as in the commensal bacterial composition in the gut, with higher abundance of Proteobacteria in mice carrying the variant. These observations are in line with the previously described links of the mitochondrial complex III gene with obesity and metabolic diseases in humans. Given that these functional changes by the G variant at m.15124 in the *mt-Cytb* are already present in young mice that were kept under normal condition, it is plausible that the m.15124A>G variant is a disease susceptibility modifier to the diseases induced by additional stressors, i.e., dietary and/or aging stress, and that the variant results in the higher incidence of clinical diseases presentation in C57BL/6J-mt^129S1/SvlmJ^ than C57BL/6J mice. Thus, mtDNA variants could be potential biomarkers to evaluate the healthspan.

## 1. Introduction

Mitochondria are one of the major sources of cellular energy in the form of adenosine triphosphate (ATP) and are known as signaling platforms, e.g., by producing reactive oxygen species (ROS) [1,2]. Thus, changes of mitochondrial function lead to the alteration of cellular differentiation and functions and consequently result in a variety of pathological conditions, including metabolic diseases, chronic inflammatory disease and aging [3]. Mitochondria contain over 1500 proteins that are encoded by the nuclear and the mitochondrial genome (mtDNA). The mtDNA is present in several copies in a mitochondrion, with the size of approximately 16 kilobases, and encodes only 37 genes, i.e., 13 oxidative phosphorylation (OXPHOS) complex protein-coding genes, 22 transfer RNA genes and two ribosomal RNA genes in mammals [4,5,6]. The mtDNA is polymorphic, and variations in the mtDNA link to mitochondrial functional alterations. The mtDNA variants, which are clinically relevant in humans, are categorized into three groups: (i) Recent maternally inherited deleterious mutations, (ii) ancient adaptive polymorphisms, and (iii) somatic mutations that accumulate during development and in tissues with age [7]. Deleterious mutations that cause classical mitochondrial diseases, such as Leigh syndrome and Leber’s hereditary optic neuritis, are well described. Ancient adaptive polymorphisms are utilized to determine mitochondrial haplogroup ancestry. Moreover, it has been reported that several ancient polymorphisms in the mtDNA have been associated with different susceptibility to common diseases, including chronic inflammation and metabolic diseases that result from mitochondrial dysfunctions as described above [3]. One of such examples is the polymorphism in the mitochondrially encoded cytochrome b gene (*MT-CYB*), which has been reported to be associated with middle-aged obesity in the Japanese population though the functional consequence of the variation in the *MT-CYB* gene that is yet to be elucidated [8]. 

During the last few decades, obesity has been spreading like an epidemic across the world. The World Health Organization reported that, globally, 1.9 billion adults were overweight and over 650 million were obese in 2016 [9]. Obesity itself is known to cause a persistent low-grade inflammatory state in metabolic tissues by producing pro-inflammatory mediators, such as leptin, interleukin (IL)-6, and TNF-α, and this condition is termed as “metaflammation”, which predisposes obese individuals to cardiovascular diseases, metabolic diseases, such as type 2 diabetes (T2DM), and chronic inflammatory disorders [10]. Therefore, identifying pathways controlling obesity is urgently warranted and has an impact on our society both medically and economically.

To date, mitochondrial dysfunctions [11,12,13] and dysbiosis in the gut [14,15,16] have been well-documented as pathologically contributing factors to metabolic disorders, including obesity. Recently, our group has shown experimental evidence that the natural variants in the mtDNA, which cause alterations in mitochondrial function, are associated with shifts in composition of gut microbiota in mice [17].

In this study, we found that mice carrying a maternally inherited polymorphism in the *mt-Cytb* in complex III (m.15124A>G, Ile-Val) demonstrated higher body weight at 12 months of age, and we investigated the functional consequences of the polymorphism on mitochondrial functions and the gut microbiota, which are known to be linked with obesity and age-related diseases. 

## 2. Results

### 2.1. Mice Carrying a Variant in the mt-Cytb Gene in the Mitochondrial Complex III Are Susceptible to Obesity

**Figure 1:**
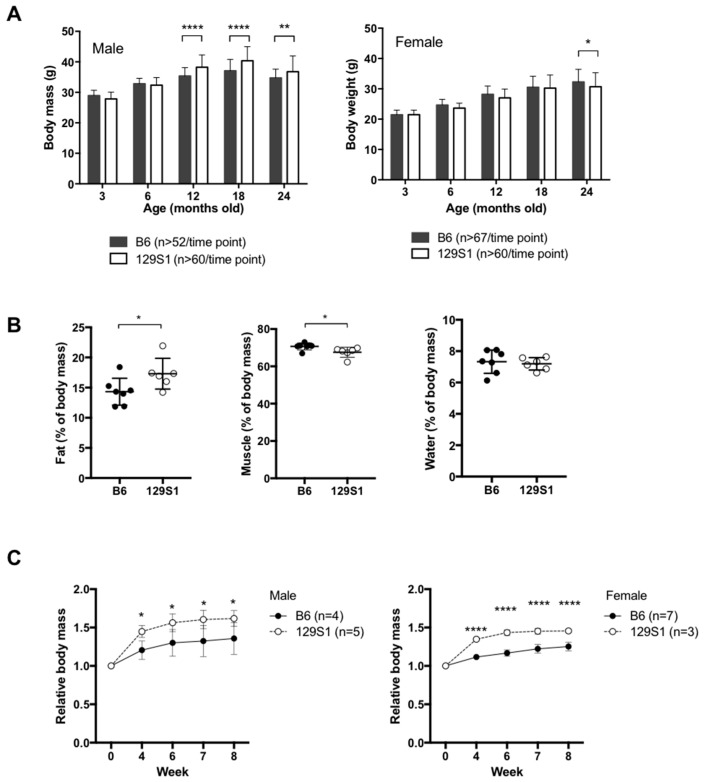
Mice carrying a single variant in the mitochondrially encoded cytochrome b gene (m.15124A>G) are susceptible to middle-aged obesity. (**A**) Body weight of male mice measured at different ages. Values were obtained from different individuals at each time point. ** *p* < 0.01, **** *p* < 0.0001; two-way ANOVA. (**B**) Body composition (fat, muscle, and water) was analyzed in young (3 months old) C57BL6J (*n* = 7) and C57BL6J-mt^129S1/SvlmJ^ mice (*n* = 6) using NMR. * *p* = 0.042 (fat), * *p* = 0.0295 (muscle) and *p* = 0.6881 (water); unpaired *t*-test. (**C**) High fat diet (with 60% energy from fat)-induced weight gain and was evaluated in both male and female C57BL/6J and C57BL/6J-mt^129S1/SvlmJ^ mice. The values presented in the figures are the body weight relative to that at week 0. * *p* < 0.05, **** *p* < 0.0001; two-way ANOVA. B6; C57BL/6J, 129S1; C57BL/6J-mt^129S1/SvlmJ^.

We evaluated the body weight of C57BL/6J-mt^129S1/SvlmJ^ and C57BL/6J mice, which were kept under normal condition (chow diet, ad libitum, 12 h light/dark cycle), at 3, 6, 12, 18, and 24 months of age. C57BL/6J-mt^129S1/SvlmJ^ mice carrying the m.15124A>G variant in the *mt-Cytb* gene exhibited significantly larger body weight at the age of 12 months compared with age-matched C57BL/6J mice. This phenotype was particularly observed in males but not females (Figure 1A). We also recorded any pathological phenotype observed during the lifespan of the mice or at autopsy when moribund. A significantly higher incidence of ulcerative dermatitis (*p* = 0.0081, Fisher’s exact test) and arthritis (*p* = 0.0019, Fisher’s exact test) as well as a trend of higher tumor burden (*p* = 0.2055, Fisher’s exact test) were observed in C57BL/6J-mt^129S1/SvlmJ^ mice compared with C57BL/6J mice (Figure S1A). The lifespan of C57BL/6J-mt^129S1/SvlmJ^, however, did not differ from that of C57BL/6J mice despite the higher body weight and higher incidence of age-related diseases (Figure S1B). 

To evaluate the predisposition to middle-aged obesity in C57BL/6J-mt^129S1/SvlmJ^ mice, we analyzed the body composition of young C57BL/6J-mt^129S1/SvlmJ^ and C57BL/6J mice using nuclear magnetic resonance (NMR). The proportion of fat mass in C57BL/6J-mt^129S1/SvlmJ^ was significantly higher compared with that in C57BL/6J mice, suggesting that the single natural variant in complex III leads to the alteration in body fat mass before exhibiting clinical body weight difference (Figure 1B). Indirect calorimetric cage analysis of the mice revealed that the C57BL/6J-mt^129S1/SvlmJ^ mice showed a tendency of less energy expenditure compared to C57BL/6J mice and a trend of higher reliance on fat utilization as an energy source when resting, but food and water intake was comparable (Figure S1C). Next, we examined whether such change in composition could affect the susceptibility to metabolic stress, i.e., dietary challenge, in the mice carrying the natural variant in the *mt-Cytb*. Therefore, we fed the mice with a high fat diet (HFD, w/60% energy from fat) and evaluated the body weight over 8 weeks. Both male and female C57BL/6J-mt^129S1/SvlmJ^ mice gained more weight compared with sex-matched C57BL/6J mice (Figure 1C). Thus, the G variant at m.15124 in the *mt-Cytb* negatively impacted the healthspan in mice, i.e., higher incidence of middle-aged obesity and naturally occurring age-related inflammatory diseases, as well as higher susceptibility to diet-induced obesity, without affecting their overall lifespan under the normal housing condition.

### 2.2. The Natural Single Variant at m.15124 in the mt-Cytb Gene in Mitochondrial Complex III Leads to the Higher Respiration Ex Vivo

**Figure 2:**
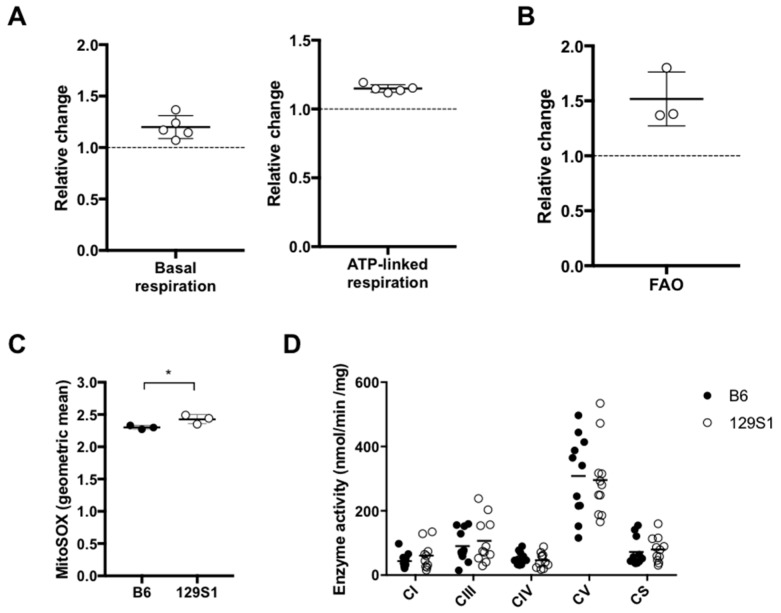
Mitochondrial functions in C57BL/6J-mt^129S1/SvlmJ^ mice compared with the wild-type C57BL/6J mice. (**A**) Relative change of basal respiration and ATP-linked respiration of primary lymphocytes from C57BL/6J-mt^129S1/SvlmJ^ mice compared to those from C57BL/6J mice. Oxygen consumption levels in murine primary lymphocytes were determined using the Seahorse XF24 analyzer. Basal respiration and ATP-linked respiration were calculated based on oxygen consumption rate (OCR) values as previously described [18]. In each experiment, 10 replicates per mouse per strain were tested (*n* = 1/strain used in one experiment). All values of one experiment were normalized with the average value of 10 replicates from the C57BL/6J mouse of that experiment to determine the relative change of lymphocyte respiration in C57BL/6J-mt^129S1/SvlmJ^ compared to C57BL/6J in each experiment. All data from five independent experiments were analyzed together. *n* = 5/strain, *p* = 0.01507 (basal respiration), *p* = 0.00005 (ATP-linked respiration); one sample *t*-test. (**B**) Relative change of fatty acid oxidation measured in primary lymphocytes of C57BL/6J-mt^129S1/SvlmJ^ compared with C57BL/6J. Oxygen consumption levels when palmitate is added as a substrate, i.e., fatty acid oxidation, were determined in murine primary lymphocytes using the Seahorse analyzer. *n* = 3/strain, *p* = 0.06750; one sample *t*-test. (**C**) Mitochondrial superoxide levels in murine primary lymphocytes were measured by flow cytometry using MitoSOX. Geometric mean of MitoSOX signal on Annexin V^low^ viable cell population was taken as the mitochondrial superoxide levels. *n* = 3/strain, * *p* = 0.0465, unpaired *t*-test. (**D**) Mitochondrial oxidative phosphorylation (OXPHOS) complex enzyme activities in liver mitochondria isolated from mice. No significant difference was observed between the strains. *n* = 11/strain, B6, C57BL/6J; 129S1, C57BL/6J-mt^129S1/SvlmJ^.

Next, we explored the functional consequence of the mitochondrial polymorphism m.15124A>G in the *mt-Cytb* gene. To evaluate the mitochondrial respiration capacity, oxygen consumption rate was measured in mesenteric lymphocytes prepared from C57BL/6J-mt^129S1/SvlmJ^ and C57BL/6J mice using a Seahorse XF24 flux analyzer. The relative changes of basal respiration and ATP-linked respiration in lymphocytes from the C57BL/6J-mt^129S1/SvlmJ^ compared to those from C57BL/6J mice were significantly greater than 1 (basal respiration, *p* = 0.01507; ATP-linked respiration, *p* = 0.00005; one sample *t*-test; Figure 2A), indicating that basal respiration and ATP-linked respiration in C57BL/6J-mt^129S1/SvlmJ^ were significantly higher than those in C57BL/6J mice. Relative changes of maximal respiration, spare capacity, and non-mitochondrial respiration values were similar to 1 (*p* > 0.05; one sample *t*-test), indicating that these parameters were comparable between the two strains. When palmitate was used as a substrate, relative change of the respiration in the primary lymphocytes from C57BL/6J-mt^129S1/SvlmJ^ compared to those from C57BL/6J mice was larger than 1 (*p* = 0.06750, one sample *t*-test; Figure 2B), suggesting that the C57BL/6J-mt^129S1/SvlmJ^ mice exhibited a tendency of higher levels of fatty acid β-oxidation (FAO) than C57BL/6J mice. We next measured the levels of mitochondrial superoxide, byproduct of the OXPHOS respiration, in the lymphocytes using the MitoSOX probe by flow cytometry. The lymphocytes prepared from C57BL/6J-mt^129S1/SvlmJ^ mice demonstrated significantly higher levels of mitochondrial superoxide when compared with those from C57BL/6J (Figure 2C; *p* = 0.0465, unpaired *t*-test). In addition, OXPHOS complex enzyme activity was measured in liver mitochondria isolated from the mice. The activities of complex I, III, IV and V, as well as citrate synthase, were comparable between the strains (Figure 2D). The levels of OXPHOS protein abundance in the liver tissues were comparable between the strains (Figure S2). These data suggest that the mice carrying the G variant at m.15124 in the *mt-Cytb* in complex III exhibit increased mitochondrial respiration without altering the OXPHOS complex enzyme activities and protein abundance, compared with wild-type C57BL/6J mice carrying the A variant at the position.

### 2.3. C57BL/6J-mt^129S1/SvlmJ^ Mice Exhibit a Distinct Pattern of Gut Microbiota Composition

There is growing evidence that composition of commensal bacteria in the gut has a strong impact on obesity in humans and mice [14,19]. Therefore, we analyzed the gut microbiota of C57BL/6J-mt^129S1/SvlmJ^ and C57BL/6J mice using bacterial 16S ribosomal RNA gene next-generation sequencing (V1-V2 region) at different ages, i.e., young, middle, and old.

**Figure 3:**
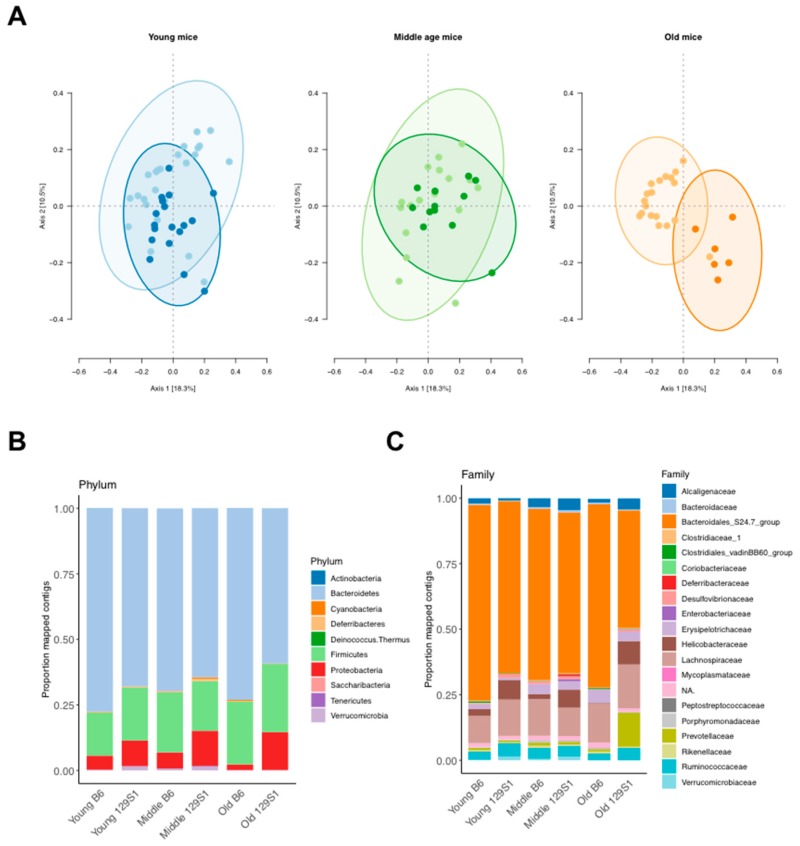
Commensal bacterial composition in the gut differs between C57BL/6J-mt^129S1/SvlmJ^ carrying the G variant at m.15124 in the *mt-Cytb* gene and wild-type C57BL/6J mice at the phylum and the family levels. (**A**) Beta diversity of gut microbiota at different age: Young (3 to 6 months old), middle age (10 to 14 months old), and old (24 to 26 months old). Ellipses correspond to the 95% confidence interval of the data. For better visualization, beta diversities are depicted separately for each age group, while the analysis was performed using all groups together (cf. Figure S3B for all beta diversities plotted together). Lighter-colored dots indicate wild-type C57BL/6J mice, while the darker-colored dots indicate C57BL/6J-mt^129S1/SvlmJ^ mice. (**B**) Phylum level. (**C**) Family level.

Alpha diversity of the gut microbiota from each group showed no significant difference (*p* > 0.05, Figure S3A), suggesting that the richness and evenness of gut microbial species between C57BL/6J-mt^129S1/SvlmJ^ and C57BL/6J mice were comparable. Beta diversity, which represents the overlap or dissimilarity between multiple populations, was significantly different when using Bray–Curtis dissimilarity between strains at all age groups (adonis test: *r*^2^_age_ = 0.05870, *p*_age_ = 0.0001; *r*^2^_strain_ = 0.05655, *p*_strain_ = 0.0001; *r*^2^_strain:age_ = 0.06154, *p*_strain:age_ = 0.0001; Figure 3A and Figure S3B), indicating the proportion of bacterial species was significantly different between the two strains at different ages.

Next, the inter-strain difference of commensal bacteria composition was evaluated (Figure 3B,C). The most abundant phyla in each group were Bacteroidetes and Firmicutes in all groups, as expected. Significant differences between the strains were identified as shown in Table 1. In all age groups, Proteobacteria was significantly more abundant in C57BL/6J-mt^129S1/SvlmJ^ compared to C57BL/6J mice. At the family level, *Desulfovibrionaceae* was more abundant in young and old C57BL/6J-mt^129S1/SvlmJ^ mice than C57BL/6J mice but not in the middle-aged mice. These data confirmed that the polymorphism at m.15124A>G in the *mt-Cytb* gene in mitochondrial complex III resulted in differential gut microbiota composition in mice, which is in line with our previous report [17].

The ratio of Firmicutes to Bacteroidetes, which was previously reported as an indicator of obesity [15,20], remained similar between the strains, irrespective of age (Mann–Whitney *U* test: *p*_young_ = 0.1841, *p*_middle_ = 0.5558, *p*_aged_ = 0.2736; uncorrected *p*-values).

## 3. Discussion

Mitochondrial genome polymorphisms are believed to have arisen during our ancestors’ migration across continents, primarily to adapt to the different environments, such as climate and nutritional availability, because such variations result in mitochondrial functional alterations [3,7]. For example, those who lived under restricted food conditions had to facilitate efficient energy production capacity from limited nutrition. Therefore, those who did not carry such variations in the mtDNA and could not adapt to the given environment had to struggle in the given environment and were potentially forced to migrate to other habitats to survive. 

Over the last few decades, our lifestyle has changed dramatically, e.g., dietary habit and a low physical activity are more common, and this has contributed to the global increase of overweightness and obesity [21]. As hypothesized by Wallace in his reviews [3,22], dietary availability used to be periodic according to the seasons. Cells adapt their OXPHOS depending on the supply of plant-derived carbohydrate nutrition, down-regulating OXPHOS when plant-derived glucose to generate ATP by glycolysis was sufficiently available. Excess energy is stored as fat in energy-storage tissues, e.g., white adipose tissue. When plant calories were not available in the non-growing season, fat from the energy-storage tissues was utilized by fatty acid oxidation to fuel mitochondria, and OXPHOS was up-regulated to generate ATP to survive through the season. In contrast, nowadays, calories are constantly available, resulting in a constant down-regulation of OXPHOS, fat storage and ATP turnover due to less physical activity. Thus, the advantages of mtDNA variability towards different environments and nutrient availability may now result in obesity for individuals with mtDNA variants that down-regulate OXPHOS to efficiently utilize glucose and promote fat storage. Therefore, it is logical that functional consequences of specific natural mtDNA variants could result in a pathological outcome when carriers of the variants are exposed to different environments. Needless to mention, the nuclear genome background also affects the functional consequence of mtDNA variations [23,24], and mtDNA variations could modulate the impact of nuclear DNA mutations, as previously reported [25].

In this study, we found spontaneous middle-aged obesity phenotype in male C57BL/6J-mt^129S1/SvlmJ^ mice under normal housing condition, leading us to further investigate the impact of the mitochondrial genome polymorphism, m.15124A>G on other phenotypes in mice. In addition to the middle-aged obesity, C57BL/6J-mt^129S1/SvlmJ^ mice exhibited higher susceptibility to diet-induced obesity and higher incidence of age-related diseases in C57BL/6J-mt^129S1/SvlmJ^ mice compared with C57BL/6J. Most importantly, C57BL/6J-mt^129S1/SvlmJ^ mice did not present any clear pathological alterations, i.e., disease-free, until they reached approximately 12 months old, the age equivalent to the 30s to 40s in humans [26]. It is noteworthy that relatively mild changes in body composition, metabolism, mitochondrial functions, and gut microbiota composition were already present in C57BL/6J-mt^129S1/SvlmJ^ mice at a younger age. The m.15124A>G variant is derived from the strain 129S1/SvlmJ. By repeated backcrossing with male C57BL/6J mice over 12 generations, the C57BL/6J-mt^129S1/SvlmJ^ strain was generated [27]. Prior studies reported an obesity phenotype in the 129S1/SvlmJ strain and C57BL/6J mice. Interestingly, the results are contradictory. Su et al. reported that 129S1/SvlmJ mice were more susceptible to atherogenic diet-induced obesity than C57BL/6J [28], while Ussar et al. published that the 129S1/SvlmJ strain was more resistant to high fat diet-induced obesity than C57BL/6J [29]. Despite these reports describing different metabolic phenotypes, it is clear that the metabolically related pathways are distinct between the original two strains, 129S1/SvlmJ and C57BL/6J, suggesting an involvement of m.15124A>G in obesity.

When mitochondrial functions were evaluated, higher oxygen consumption, a tendency of higher FAO levels, and greater levels of ROS were found in C57BL/6J-mt^129S1/SvlmJ^ mice compared with C57BL/6J. The observed trend in FAO levels between the strains, i.e., higher levels of FAO in C57BL/6J-mt^129S1/SvlmJ^ mice than C57BL/6J mice, is consistent with the calorimetric cage analysis data, which showed that the former tended to utilize more fat as an energy source in the resting period (i.e., between 6:00 and 18:00). Nicotinamide adenine dinucleotide (NADH), flavin adenine dinucleotide (FADH_2_), and acetyl-CoA are generated by FAO and fuel the OXPHOS respiration (the former two products do so directly, and the latter one does so via tricarboxylic acid cycle) [30], indicating that the higher OXPHOS respiration may be the result of the increased FAO levels in C57BL/6J-mt^129S1/SvlmJ^ mice. These data are in line with previous reports about the link between these mitochondrial dysfunctions and metabolic diseases, including obesity [11,31,32,33]. The gut microbiota analysis demonstrated a distinct commensal bacterial composition pattern in C57BL/6J-mt^129S1/SvlmJ^ mice compared with C57BL/6J mice at all age groups tested in this study. At the phylum level, Proteobacteria, which are reportedly related to metabolic diseases including obesity and inflammatory disorders [34,35,36], were found to be more abundant in C57BL/6J-mt^129S1/SvlmJ^ mice than wild-type C57BL/6J mice. Furthermore, *Christensenellaceae*, *Coriobacteriaceae* and *Desulfovibrionaceae*, which were also known to link with obesity and other metabolic dysfunctions [37,38,39], were more abundant in young C57BL/6J-mt^129S1/SvlmJ^ mice than age-matched C57BL/6J mice, indicating the G variant at m.15124 in the *mt-Cytb* shifts gut microbiota composition toward the one that makes the host more susceptible to metabolic and inflammatory disorders in mice.

These changes in mitochondrial functions and gut microbiota composition caused by the G variant in m.15124 in the *mt-Cytb* in complex III already occurred when C57BL/6J-mt^129S1/SvlmJ^ mice were young, although such alterations were subclinical. Dietary stress or natural aging stimuli triggered the onset of pathological phenotypes in both C57BL/6J-mt^129S1/SvlmJ^ mice and C57BL/6J mice but resulted in higher susceptibility only in C57BL/6J-mt^129S1/SvlmJ^ mice. In fact, the difference in gut microbiota composition between the strains was more prominent in aged mice than young mice. Composition of the gut microbiota reportedly differs from that of younger individuals in humans and mice [40,41,42]. Aging is a progressing phenomenon [43], while the m.15124A>G variant is stable. Therefore, it is hypothesized that aging may amplify the difference in the gut microbiota between C57BL/6J-mt^129S1/SvlmJ^ and C57BL/6J that was caused by the m.15124A>G variant in *mt-Cytb*. Thus, the difference observed in young mice is mainly due to the mitochondrial variant, and the difference between the two strains may become more distinct due to the synergetic effect of progressing age in addition to the already existing mtDNA variant. This finding shows that mtDNA polymorphisms are a modifier not only of autosomal diseases [25] but also of environmental- and aging stress-induced pathological conditions. 

The impact of the m.15124A>G variant in *mt-Cytb* on the body weight at the age of 12 months was only observed in males and not in females. On the other hand, the incidence of spontaneous age-associated pathological phenotypes, i.e., ulcerative dermatitis and arthritis, demonstrated an inverse incidence between males and females, yet were always increased in C57BL/6J-mt^129S1/SvlmJ^ compared to C57BL/6J mice. Overall, the incidence of ulcerative dermatitis was lower in males, with a less pronounced effect of the variant on males and a significant effect of that on females (males, 3.90% in C57BL/6J and 12.20% in C57BL/6J-mt^129S1/SvlmJ^, *p* = 0.0563, Fisher’s exact test; females, 16.09% in C57BL/6J and 28.71% in C57BL/6J-mt^129S1/SvlmJ^, *p* = 0.0399, Fisher’s exact test). The incidence of arthritis was higher in males than in females, with a significant impact of the variant on males and a less pronounced effect on females (males 7.23% in C57BL/6J and 18.81% in C57BL/6J-mt^129S1/SvlmJ^, *p* = 0.0297, Fisher’s exact test; females, 0% in C57BL/6J and 4.72% in C57BL/6J-mt^129S1/SvlmJ^, *p* = 0.0653, Fisher’s exact test). This could be due to the sex-specific incidence of the diseases. These two spontaneous diseases have been previously described for their sex-specificity. C57BL/6J males are more often affected by spontaneous arthritis than females [44,45], while C57BL/6J females exhibit the higher incidence of spontaneous ulcerative dermatitis than males [46,47], suggesting that the impact of the m.15124A>G variant, which appears to be sex-specific at first glance, seems to affect both sexes, but the effect is more pronounced in the previously described susceptible sex. Concerning the body weight gain, the male C57BL/6J mice are known to be more susceptible to diet-induced obesity than females [48,49]. Therefore, the body weight gain observed in male C57BL/6J-mt^129S1/SvlmJ^ mice after 12 months of age may be enhanced by the *mt-Cytb* variant.

In summary, we report experimental evidence that a maternally inherited single natural variation in the mitochondrial genome, m.15124A>G in complex III, is a predisposing factor to age-related metabolic and inflammatory disorders by altering metabolism, mitochondrial functions, and gut microbiota composition already in earlier life, suggesting such non-deleterious and adaptive polymorphisms in the mtDNA as a potential biomarker of the healthspan in humans.

## 4. Materials and Methods

### 4.1. Mice and Husbandry

C57BL/6J mice (Stock no.: 000664) were obtained from the Jackson Laboratory (Bar Harbor, ME, USA) and bred in the animal facility of the University of Lübeck. The conplastic strain C57BL/6J-mt^129S1/SvlmJ^ was generated as previously described [27]. The mutations in the mtDNA of the C57BL/6J-mt^129S1/SvlmJ^ and C57BL/6J mice are listed in Table S1. For genotype nuclear genome of both C57BL/6J-mt^129S1/SvlmJ^ and C57BL/6J, we used the MegaMUGA Mouse Universal Genotyping Array (77,808 single-nucleotide polymorphisms (SNPs)) as described previously [24,50]. More than 99.9% of SNPs in C57BL/6J-mt^129S1/SvlmJ^ mice were identical to those of C57BL/6J (Table S2).

The C57BL/6J-mt^129S1/SvlmJ^ strain was maintained by repeatedly backcrossing female C57BL/6J-mt^129S1/SvlmJ^ with male C57BL/6J mice, which were randomly selected from the C57BL/6J colony maintained in the same breeding facility room. With this breeding strategy, there was ample opportunity for microbiota to be homogenized between the strains, i.e., genetic factors are not confounded by separate housing.

### 4.2. Animal Experiments

Body weight determination and feeding experiment: Four-week-old mice were fed with a high fat diet (HFD, with 60% energy from fat, C1090-60, Altromin, Lage, Germany) over 8 weeks. Body weight was measured.

Lifespan study and determining age at death: 87 female C57BL/6J-mt^129S1/SvlmJ^, 87 female C57BL/6J, 84 male C57BL/6J-mt^129S1/SvlmJ^, and 68 male C57BL/6J mice were used to evaluate their lifespan, assuring a statistical power to detect a 10% difference in lifespan at a significance level of 0.05 and a power of 0.8 using G*Power [51]. Daily inspection of mice, the determination of moribund condition and the best available lifespan of mice, and the autopsy of moribund mice were performed as previously described [24,50,52]. 

Body composition assessment by nuclear magnetic resonance (NMR): Lean and fat mass in mice were determined using the benchtop NMR analyzer Minispec LF90 (Bruker Biospin MRI GmbH, Ettlingen, Germany), as previously described [53].

Indirect calorimetric cage analysis: Oxygen consumption, carbon dioxide production, food and water intake, and locomotor activity were continuously monitored using an open-circuit indirect calorimetry system (PhenoMaster System^TM^, TSE, Bad Homburg, Germany) as previously described [50]. 

Animal use and all procedures used in this study were approved by the local authority of the Animal Care and Use Committee (V242.7224.122-5, and 94-7/13, Kiel, Germany) and performed in accordance with the relevant guidelines and regulations by certified personnel. 

### 4.3. Mitochondrial Functional Assays

Seahorse analysis of primary lymphocytes: Oxygen consumption rate (OCR, in pmol/min) was measured using the Seahorse XF24 flux analyzer (Agilent, North Billerica, USA), as previously described [17,54]. Freshly prepared lymphocytes from murine mesenteric lymph nodes were plated onto a CellTak-coated 24-well Seahorse XF-24 assay plate at 1.5 × 10^6^ cells per well. For the Mito Stress test, measurement was started after replacing the medium into XF Mito Stress assay buffer, i.e., DMEM without bicarbonate, supplemented with 4.5 g/L D-glucose, 2 mM L-glutamine, 1 mM pyruvate, pH 7.4. After baseline determination, 1 µM oligomycin was added to inhibit the ATP synthase, thereby determining oxygen consumption levels used for ATP-linked respiration and proton leak. Next, 0.4 µM FCCP was added to uncouple the mitochondrial membrane potential, allowing maximal electron flux to determine the maximal respiration and spare capacity. Finally, a mixture of 1 µM antimycin A and 1 µM rotenone was administrated to completely block mitochondrial electron transport and determine the non-mitochondrial oxygen consumption levels. Calculation of basal oxygen consumption, ATP-linked respiration, maximal respiration, spare capacity, and non-mitochondrial respiration values were performed according to the previous report [18]. Fatty acid β-oxidation (FAO) was measured as previously reported with slight modifications [55,56]. The assay was performed in the fatty acid oxidation buffer, i.e., KHB buffer (111 mM NaCl, 4.7 mM KCl, 1.25 mM CaCl_2_, 2mM MgSO_4_, 1.2 mM NaH_2_PO_4_) supplemented with 2.5 mM glucose, 0.5 mM carnitine, and 5 mM HEPES, pH 7.4 at 37 °C. After baseline determination, 200 µM palmitate was added, and oxygen consumption levels, by utilizing fatty acid as an energy source, were measured. A mixture of 2 µM antimycin A and 2 µM rotenone was injected to block mitochondrial respiration. All chemicals used in this assay were purchased from Sigma-Aldrich Chemie GmbH (Munich, Germany), unless specified.

Mitochondrial superoxide assay using flow cytometry: Mitochondrial superoxide was assessed in primary lymphocytes immediately after the preparation. Cells were washed with 2% fetal bovine serum in PBS and stained with 5 µM MitoSOX Red (Invitrogen, MA, USA) for 10 min at 37 °C, followed by Annexin V-APC (ThermoFisher Scientific, MA, USA) staining for 10 min at room temperature, and measured using FACSCalibur (BD Science, Heidelberg, Germany). Data were analyzed using the FlowJo software, and geometric means of the MitoSOX signal in the Annexin V low lymphocyte population were taken as the value of mitochondrial superoxide. 

Mitochondrial OXPHOS complex enzyme activity assay: Mitochondria were prepared from freshly removed liver of C57BL/6J-mt^129S1/SvlmJ^ and C57BL/6J mice and tested for the mitochondrial OXPHOS complex enzyme activities, as previously described [24,50]. In brief, assays were performed in 200 µL/well in a 96-well plate at 37 °C (except citrate synthase, which were performed at 30 °C), using a Tecan Infinite 200 PRO series spectrophotometer (Tecan Group Ltd., Männedorf, Switzerland).

Mitochondrial OXPHOS complex protein determination by Western blotting: Frozen liver tissues were lysed in RIPA buffer, and the supernatant was used for Western blotting, as previously described [50].

### 4.4. 16S rRNA Gene Next-Generation Sequencing

Fecal samples were collected from 60 C57BL/6J mice (24 young, 17 middle-aged, and 19 old individuals) and 36 C57BL/6J-mt1^29S1/SvlmJ^ mice (18 young, 12 middle age, and 6 old individuals). Age groups were defined as young: 3 to 6 months old, middle age: 10 to 14 months old, and old: 24 to 26 months old.

DNA extraction from fecal samples and 16S rRNA gene sequencing was conducted as previously described [17]. In brief, the 16S rRNA gene was amplified using uniquely barcoded primers flanking the V1 and V2 hypervariable regions (27F-338R) with fused MiSeq adapters, and the PCR products were processed for the library preparation. The library was sequenced on the Illumina MiSeq platform with v3 chemistry using 2 × 300 cycles.

### 4.5. MiSeq Data Analysis and Taxonomic Classification

Raw sequencing reads were demultiplexed using bcl2fastq2 conversion software distributed by Illumina. Afterwards, reads were merged using fastq_mergepairs from VSEARCH package [57] version 1.9.9 with a maximum number of mismatches of 12 and the merged reads (contigs) between 270 and 330 bp long. Next, a quality filter step was applied as implemented in the fasta_filter command from the USEARCH [58] version 8.1.1861 with the expected numbers of errors set to 0.5. Chimeras were identified by uchime_ref (USEARCH) with the RDP Gold database version 9 as a reference database and were removed from the contig set. Subsequently, unique sequences were identified (derep_fulllength; VSEARCH) and sequences were clustered into operational taxonomic units (97% identity) using UPARSE [59] version 8.1.1861.

Taxonomy was assigned to genus level for all non-chimeric sequences using Mothur [60] version 1.36.1 and the SILVA database version 123 with 80% bootstrap support (1000 iterations). Sequences of non-bacterial origin were removed from the dataset, and remaining sequences were aligned to the 16S rRNA V1-V2 region against the SILVA database. Finally, FastTree [61] version 2.1.4 was utilized to construct a phylogenetic tree with a generalized time-reversible (GTR) substitution model and the gamma option to rescale branch length. The resulting tree was rooted using the midpoint method for rooting. OTUs present in only one individual with one contig (singletons) were removed, as well as OTUs with unclassified Phylum assignments. Next, 12,800 contigs were selected randomly per stool sample. This resulted in 2179 OTUs for further analysis. 

Alpha and beta diversity analysis: Two alpha diversity indices (Chao1, Shannon) were estimated using the vegan [62] package version 2.5-3. Bray–Curtis dissimilarity (beta diversity) was estimated using the vegan package. To investigate differences in beta diversity, non-parametric analysis of variance was used as implemented in the adonis command (vegan package). To compute significance values for each factor, 9999 permutations were run. Principle coordinate analysis was performed using the ordinate command as implemented in the phyloseq [63] package version 1.26.1. 

Statistical analyses for the microbiota study: Unless stated otherwise, all statistical analyses were performed using R [64] version 3.5.2. If not stated differently, correction for multiple testing was applied using the Benjamini–Hochberg method, as implemented in the p.adjust command (stats package for R).

## 5. Statistical Analysis for the Other Experiments

Statistical analyses for the lifespan study and other mitochondrial functional studies were performed using GraphPad Prism (GraphPad Software, San Diego, CA, USA), and statistical tests used for analysis are indicated in the figure legends.

## Figures and Tables

**Table 1:** Inter-strain differential bacterial phyla and family between C57BL/6J-mt^129S1/SvlmJ^ and C57BL/6J (*p_adj_* < 0.05).

Age Group *	Inter-Strain Differential Bacterial Phyla	Inter-Strain Differential Bacterial Family
Young	Actinobacteria, Bacteroidetes, Proteobacteria, Verrucomicrobia	*Christensenellaceae*, *Clostridiales vadinBB60 group*, *Coriobacteriaceae*, *Desulfovibrionaceae*, *Helicobacteraceae*, *Mycoplasmataceae*
Middle	Deferribacteres, Proteobacteria	None
Old	Proteobacteria	*Bacteroidales S24.7 group*, *Desulfovibrionaceae*, *Enterobacteriaceae*, *Prevotellaceae*

* Age group: Young, 3 to 6 months old; middle, 9 to 14 months old; old, 24 to 26 months old.

## Data Availability

Gut microbiota sequencing data used for this study were submitted to the European Nucleotide Archive (ENA) and are available under the accession number PRJEB31300.

## Supplementary Materials

Supplementary materials can be found at https://www.mdpi.com/1422-0067/20/9/2359/s1.
