# Supplementary material for: A Natural mtDNA Polymorphism in Complex III Is a Modifier of Healthspan in Mice
Source: Int J Mol Sci. 2019 May 13;20(9):2359. doi: 10.3390/ijms20092359 (PMC6539666; doi:10.3390/ijms20092359)
Supplement: Supplementary file 1 [file ijms-20-02359-s001.pdf]

Supplementary data

A Natural mtDNA Polymorphism in Complex III Is a Modifier of Healthspan in Mice

Supplementary figures

Supplementary figure 1

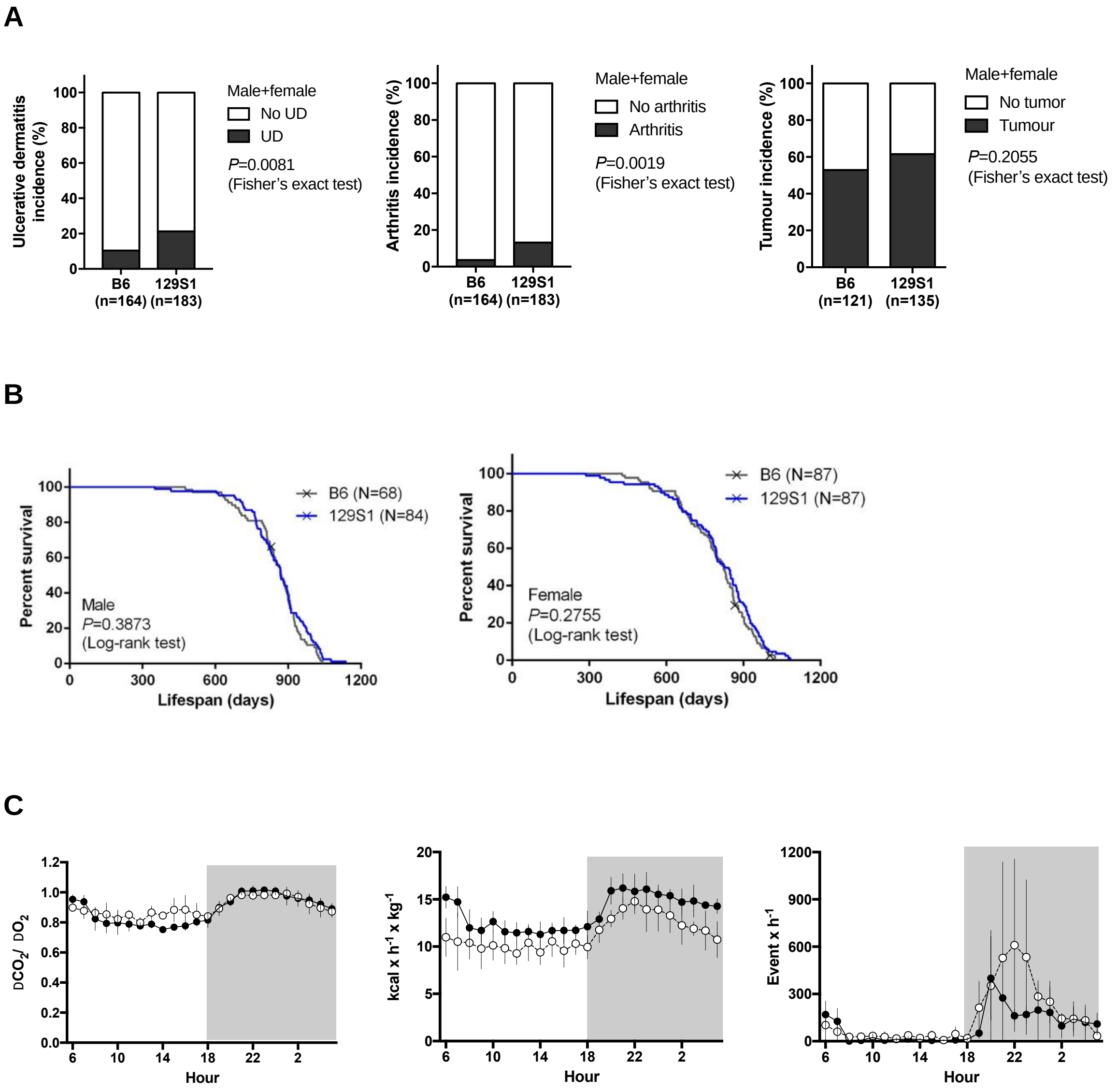

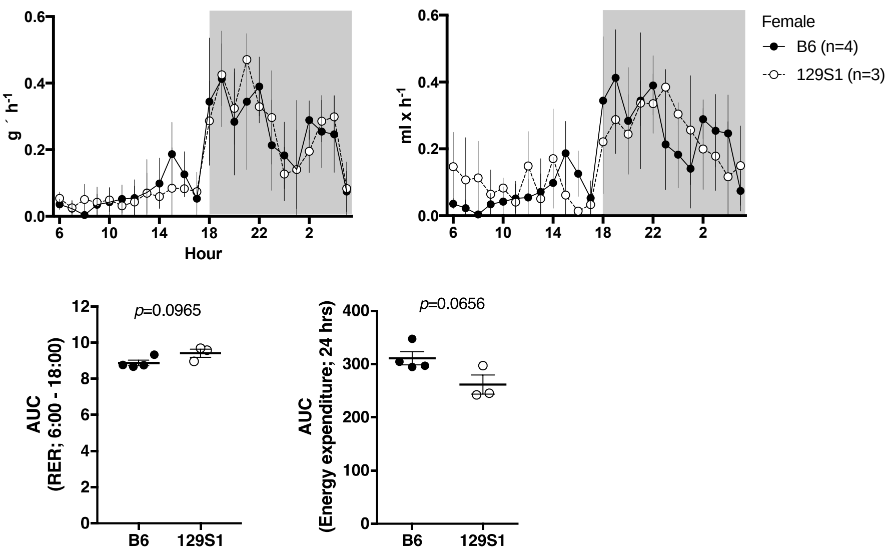

Supplementary Figure S1: The polymorphism m.15124A>G in the *mt-Cytb* gene does affect the health span but not the lifespan in mice with C57BL/6J nuclear background in this study. (**A**) Incidences of spontaneously occurring ulcerative dermatitis (UD), spontaneous arthritis, and tumor were evaluated in C57BL/6J and C57BL/6J-mt$^{129S1/SvImJ}$ mice. Of 183 C57BL/6J-mt$^{129S1/SvImJ}$ mice evaluated, 39 mice with UD and 24 with arthritis were observed, while 17 with UD and six with arthritis were found among 164 C57BL/6J mice. Tumors were identified in 83 of 135 C57BL/6J-mt$^{129S1/SvImJ}$ mice and in 64 of 121 C57BL/6J mice. (**B**) Lifespan was observed on mice, which were kept under normal condition (normal chow, 12 h light-/dark-cycle). The median survival in males was 871.0 (C57BL/6J) and 869.5 (C57BL/6J-mt$^{129S1/SvImJ}$) days, and in females this was 822.0 (C57BL/6J) and 827.0 (C57BL/6J-mt$^{129S1/SvImJ}$). (**C**) Indirect calorimetric cage analysis reveals a moderate decrease of energy expenditure in C57BL/6J-mt$^{129S1/SvImJ}$ mice (n = 3) compared with C57BL/6J mice (n = 4). Female, 3 months old. Time plot of respiratory exchange ratio (RER; $\Delta CO_2/\Delta O_2$), energy expenditure, (kcal x kg^{-1} x h^{-1}) locomotor activity measured by infrared (Event x h^{-1}), food intake (g x h^{-1}), and water intake (ml x h^{-1}). No statistical significance was observed between the strains (p = 0.3144, p = 0.0509, p = 0.3561, p = 0.9416, and p = 08204, respectively, two-way ANOVA). The area under the curve of RER during resting period (6:00 to 18:00) and that of energy expenditure values (whole day) of individual mice was plotted. p = 0.0965 (RER), p = 0.0656 (energy expenditure); unpaired t-test. B6, C57BL/6J; 129S1, C57BL/6J-mt$^{129S1/SvImJ}$.

Supplementary figure 2

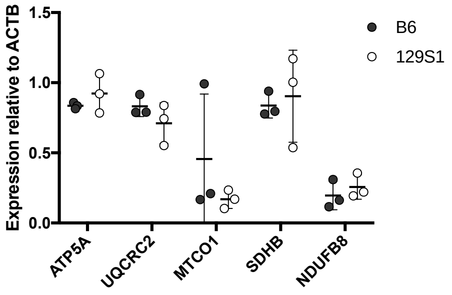

Supplementary Figure S2: Levels of OXPHOS complex protein expression in the liver tissue were comparable between C57BL/6J-mt$^{129S1/SvlmJ}$ and C57BL/6J mice. Expression of each OXPHOS protein was normalized with that of beta actin (ACTB). n = 3/strain. $p > 0.05$ for each protein; multiple *t*-test. B6, C57BL/6J; 129S1, C57BL/6J-mt$^{129S1/SvlmJ}$.

Supplementary figure 3

A

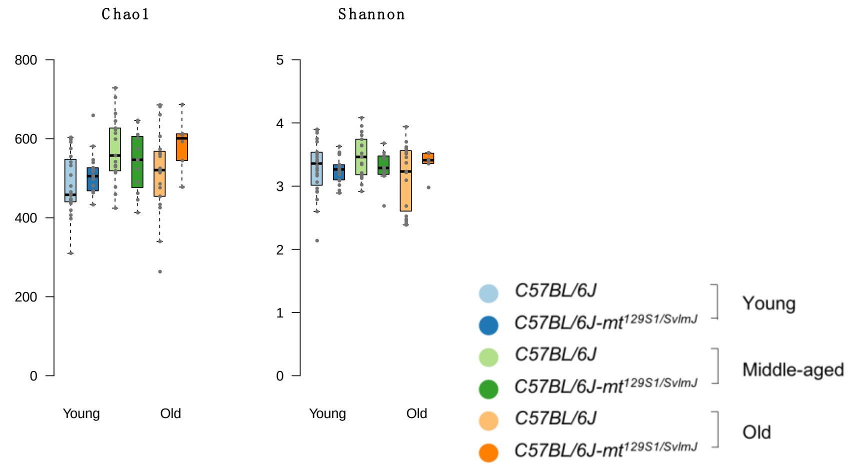

B

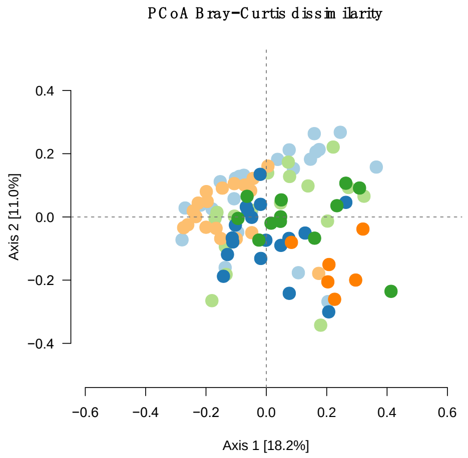

Supplementary Figure S3: Figures related to Figure 3. (**A**) Alpha diversity (Chao1 and Shannon index) was tested to evaluate the richness and evenness of gut microbiota between groups. No difference was observed in both analyses. (**B**) Beta diversity, using Bray–Curtis dissimilarity, was significantly different, as shown in the main text. A goodness-of-fit test of the PCoA revealed that all variables but age significantly correlated with difference in microbiota composition (r^2_{age} = 0.0478, p_{age} = n.s.; r^2_{strain} =

0.1093, p_{strain} = 0.0002; $r^2_{strain:age}$ = 0.2671, $p_{strain:age}$ = 0.0001). The figure presented here includes all age group samples presented in **Figure 3A**. Color codes: Lighter colors, C57BL/6J; darker colors, C57BL/6J-mt$^{129S1/SvlmJ}$, blues, young; greens, middle age; oranges, old.

Supplementary tables

Supplementary Table S1: List of mtDNA variations in C57BL/6J-mt$^{129S1/SvImJ}$ and C57BL/6J.

Position	9461	9821	15124
Strain/Gene	*mt-Nd3*	*mt-Tr*	*mt-Cytb*
C57BL/6J	T	8A	A
C57BL/6J-mt$^{129S1/SvImJ}$	C	9A	G
Amino acid change	Met-Met	-	Ile-Val

Supplementary Table S2: Nuclear genome homology of C57BL/6J-mt$^{129S1/SvImJ}$ to C57BL/6J.

Strain			**C57BL/6J**			**C57BL/6J-mt$^{129S1/SvImJ}$**					
Mouse ID			70015	70012	70004	31821	31890	34685	35865	34664	31817
Sex			M	F	F	M	F	M	M	F	F
ChrB37	PosB37	SNPs									
1	42424440	B6_rs31362610	TT	TT	TT	**TC**	TT	TT	**TC**	**TC**	TT
1	156715218	UNC010465120	TT	TT	TT	TT	**TC**	**N.D.***	TT	TT	TT
3	84097284	UNC030314030	GG	GG	GG	**GA**	GG	**GA**	**GA**	GG	GG
3	120369799	B6_03-120369799-S	AA	AA	AA	AA	**AG**	**AG**	**AG**	**AG**	AA
3	121531310	UNC030194728	CC	CC	CC	CC	**CT**	**CT**	**CT**	**CT**	CC
5	18216206	B6_rs33367397	AA	AA	AA	AA	**AG**	**AG**	**AG**	**AG**	AA
5	118166937	B6_rs29730106	AA	AA	AA	AA	**AG**	**N.D.***	AA	**AG**	**N.D.***
5	136917666	B6_rs29500641	TT	TT	TT	TT	**TG**	**TG**	**TG**	**TG**	**TG**
7	78961795	B6_rs32060039	CC	CC	CC	**CG**	**CG**	**CG**	**CG**	**CG**	CC
7	125077242	B6_rs32062246	AA	AA	AA	AA	AA	AA	AA	**AG**	AA
8	26496123	B6_rs33539160	AA	AA	AA	AA	**AG**	AA	AA	AA	AA
8	77477256	B6_rs32729089	TT	TT	TT	TT	**TA**	TT	**TA**	TT	TT
8	120161891	B6_rs32661424	CC	CC	CC	**CT**	**CT**	**CT**	**CT**	CC	CC
9	6238770	B6_09-006238770-S	AA	AA	AA	**AG**	AA	AA	**AG**	AA	AA
10	56034586	B6_rs29377979	GG	GG	GG	**GA**	GG	GG	**GA**	**GA**	**GA**
10	79915030	B6_rs29349055	AA	AA	AA	AG	AA	**AG**	AA	**AG**	**AG**
10	116077282	UNC100129834	TT	TT	TT	**TC**	TT	**TC**	TT	**TC**	**TC**
10	121868277	B6_rs29348001	AA	AA	AA	**AG**	AA	**AG**	AA	**AG**	**AG**
11	87932613	UNC20071212	CC	CC	CC	**CT**	**CT**	**N.D.***	CC	**CT**	CC
12	80337517	B6_12-080337517-S	TT	TT	TT	**TC**	**TC**	**TC**	**TC**	**TC**	**TC**
12	99208162	B6_rs29206394	AA	AA	AA	**AC**	**AC**	AA	**AC**	**AC**	**AC**
14	22151051	B6_rs31151615	TT	TT	TT	**TC**	**TC**	**N.D.***	**TC**	TT	TT
14	22662231	UNC140101805	AA	AA	AA	**AC**	**AC**	**N.D.***	**AC**	AA	AA
17	60378784	B6_rs33169019	AA	AA	AA	**AG**	**AG**	AA	AA	**AG**	**AG**
18	15323094	UNC180052085	AA	AA	AA	**AG**	**AG**	**N.D.***	**AG**	AA	AA
18	15408257	B6_rs13483221	CC	CC	CC	**CT**	**CT**	**CT**	**CT**	CC	CC
**nDNA homology to C57BL/6J (%)**			**100**	**100**	**100**	**99.98**	**99.98**	**99.98**	**99.98**	**99.98**	**99.99**

*N.D.; no data due to the genotyping errors.
